# Overcoming Challenges to Make Bacteriophage Therapy Standard Clinical Treatment Practice for Cystic Fibrosis

**DOI:** 10.3389/fmicb.2020.593988

**Published:** 2021-01-11

**Authors:** Renee N. Ng, Anna S. Tai, Barbara J. Chang, Stephen M. Stick, Anthony Kicic

**Affiliations:** ^1^School of Biomedical Sciences, The University of Western Australia, Perth, WA, Australia; ^2^Wal-yan Respiratory Research Center, Telethon Kids Institute, The University of Western Australia, Crawley, WA, Australia; ^3^Department of Respiratory Medicine, Sir Charles Gairdner Hospital, Perth, WA, Australia; ^4^Institute for Respiratory Health, School of Medicine, The University of Western Australia, Perth, WA, Australia; ^5^The Marshall Center for Infectious Diseases Research and Training, School of Biomedical Sciences, The University of Western Australia, Perth, WA, Australia; ^6^Department of Respiratory and Sleep Medicine, Perth Children’s Hospital, Perth, WA, Australia; ^7^Center for Cell Therapy and Regenerative Medicine, School of Medicine and Pharmacology, The University of Western Australia and Harry Perkins Institute of Medical Research, Perth, WA, Australia; ^8^Occupation and the Environment, School of Public Health, Curtin University, Perth, WA, Australia; ^9^Murdoch Children’s Research Institute, Melbourne, VIC, Australia; ^10^Department of Pediatrics, University of Melbourne, Melbourne, VIC, Australia

**Keywords:** bacteriophage, cystic fibrosis, lung disease, alternative therapy, animal models, antimicrobials, biofilms, regulation

## Abstract

Individuals with cystic fibrosis (CF) are given antimicrobials as prophylaxis against bacterial lung infection, which contributes to the growing emergence of multidrug resistant (MDR) pathogens isolated. Pathogens such as *Pseudomonas aeruginosa* that are commonly isolated from individuals with CF are armed with an arsenal of protective and virulence mechanisms, complicating eradication and treatment strategies. While translation of phage therapy into standard care for CF has been explored, challenges such as the lack of an appropriate animal model demonstrating safety *in vivo* exist. In this review, we have discussed and provided some insights in the use of primary airway epithelial cells to represent the mucoenvironment of the CF lungs to demonstrate safety and efficacy of phage therapy. The combination of phage therapy and antimicrobials is gaining attention and has the potential to delay the onset of MDR infections. It is evident that efforts to translate phage therapy into standard clinical practice have gained traction in the past 5 years. Ultimately, collaboration, transparency in data publications and standardized policies are needed for clinical translation.

## Introduction

Cystic Fibrosis (CF) is a life-limiting genetic disease caused by mutations to the Cystic Fibrosis Transmembrane Regulator (CFTR) gene. There are 2,000 variant mutations in the CFTR gene, however, more than 70% of people with CF carry the p.Phe508del mutation. Currently there are six classes of mutations, each with varying degrees of disruption to CFTR protein production and function (Pettit and Fellner, 2014; Lopes-Pacheco, 2016; Veit et al., 2016), correlating to varying severity of disease phenotype. The genetic defect leads to impaired water and electrolyte traffic of airway epithelial cells (AECs), tenacious airway surface liquid, impaired mucociliary clearance mucus build-up in the lungs of those afflicted due to the inability of AECs to allow chloride ions to pass into the airway surface liquid (Crawford et al., 1991; Haq et al., 2016). This leads to airway obstruction, accelerated lung function decline, reduced quality of life, and ultimately premature lung failure and death. With defective mucociliary clearance, accumulation of mucus in the airway of CF lungs occurs, creating an ideal microenvironment for the growth and persistence of bacteria. The inability to clear these bacteria allows opportunistic pathogens to establish a niche within the environment, ensuring their survival (Moreau-Marquis et al., 2008; Bjarnsholt et al., 2009; Ramsay et al., 2016).

With an increasing lifespan of CF patients due to currently available therapies and surveillance programs, isolation of multidrug resistant (MDR) bacteria from the respiratory tract has also increased. *Pseudomonas aeruginosa* (*P. aeruginosa*) remains by far the most common airway pathogen particularly amongst adults with CF. The CF Foundation has recently reported ∼17.9% of *P. aeruginosa* isolated from CF individuals in North America were MDR (Cystic Fibrosis Foundation, 2019). An earlier study conducted across CF centers in Australia found that 31 and 35% of *P. aeruginosa* isolated from a pediatric and an adult cohort, respectively, were also MDR (Smith et al., 2016). It is known that the lungs of CF individuals are prone to bacterial colonization, particularly by *P. aeruginosa*. Once colonized, the bacteria are impossible to eradicate and individuals undergo long-term antimicrobial regimes to treat and prophylactically control the rate of infection. The increasing rates of MDR infections are attributed to the prolonged use of antimicrobial drugs for both treatment and prophylaxis, and consequent gain of resistance genes and selection of hypermutator isolates (Kidd et al., 2013, 2018). Cross infection of MDR *P. aeruginosa* strains due to less stringent infection control measures had also contributed to the rapid rise in MDR rates over the past two decades (Salunkhe et al., 2005; Johansen et al., 2008; Fothergill et al., 2012; Parkins et al., 2014). Treatments for MDR pathogens are accompanied with a caveat; cumulative antibiotic burden leads to development of drug allergy and toxicity (Levison and Levison, 2009; Kalghatgi et al., 2013; Gao et al., 2017). Progressive limitation in antimicrobial treatment in CF, particularly in the aging population given the emergence of MDR organisms, antimicrobial allergy and toxicity are associated with lengthier hospitalizations, increased rates of re-admittance, and extensive treatment regimens (Baumann et al., 2003; Sansgiry et al., 2012).

The issue of MDR is now so widespread that World Health Organization (WHO) has declared the issue of antimicrobial resistance a global crisis (Shrivastava et al., 2017). The discovery pipeline into new classes of antimicrobials is also slow since it is relatively unprofitable, and innovations are unable to keep up with emerging resistance. To address this, alternative treatment methods must be explored. Treatment strategies targeting bacterial virulence and resistance have been studied extensively. Anti-virulence compounds such as quorum sensing inhibitors (Müh et al., 2006; Brackman et al., 2011; O’Loughlin et al., 2013; Soukarieh et al., 2020) and iron chelation (Moreau-Marquis et al., 2009; Parquet et al., 2018) have been found to be successful in inhibiting biofilm formation, reducing pathogenicity and increasing susceptibility to traditional antimicrobials. Strategies targeting resistance have included investigating efflux pump inhibitors (Sabatini et al., 2013; Shriram et al., 2018), anti-sense oligomers (Geller et al., 2013; Sawyer et al., 2013), immunotherapy (Feigman et al., 2018), host defense peptides (Overhage et al., 2008; de la Fuente-Núñez et al., 2015) and bacteriophages. Many of these strategies are still in exploration and validation phases and are still some way off from translating to standard clinical care practice. The vital need for a swift translation of alternative therapy into clinical use has identified bacteriophage (phage) therapy as one of the top candidates due to its successful use in humans when approved on compassionate grounds. Phage treatments are also cheaper due to shorter treatment periods, exhibit little or no toxicity, and are more effective than current antimicrobial strategies (Alemayehu et al., 2012; Agarwal et al., 2018; Oliveira et al., 2020).

## Bacteriophages and Their Therapeutic Applications to Date

Bacteriophages are viruses found ubiquitously on Earth. They are able to undergo two life cycles: lytic (lyse the bacterial host in the process of replication) or lysogenic (integrate into the genome of bacterial host). Thus, when considering these for therapeutic application, selection should primarily be limited to the use of lytic phages in order to minimize the possibility of virulence or resistance genes transfer. They were first described and observed to display lytic activity in the early 1910s by microbiologists Frederick W. Twort and Felix d’Herelle (Twort, 1914; d’Herelle, 1917) but despite their initial success, they were soon overshadowed by the introduction of antibiotics (Clokie et al., 2011). Antibiotics were considered a cheap and safe way to treat bacterial infections and their assessment was typically accompanied with well documented research that demonstrated their beneficial effects. Although resistance against penicillin emerged almost immediately after its introduction, discovery of new antibiotics maintained their primary use in the Western world. With restricted access, eastern bloc countries progressed phage therapy through to its clinical inauguration. Entities including the Eliava Institute of Bacteriophages, Microbiology and Virology (EIBMV) in Georgia and the Phage Therapy Unit at the Hirszfeld Institute of Immunology and Experimental Therapy in Poland are still operational and patients are provided personalized phage therapy for chronic infections (d’Herelle, 1931; Häusler, 2006; Kutateladze and Adamia, 2008; Chanishvili, 2012, 2016; Kutateladze, 2015; Górski et al., 2018).

**FIGURE 1:**
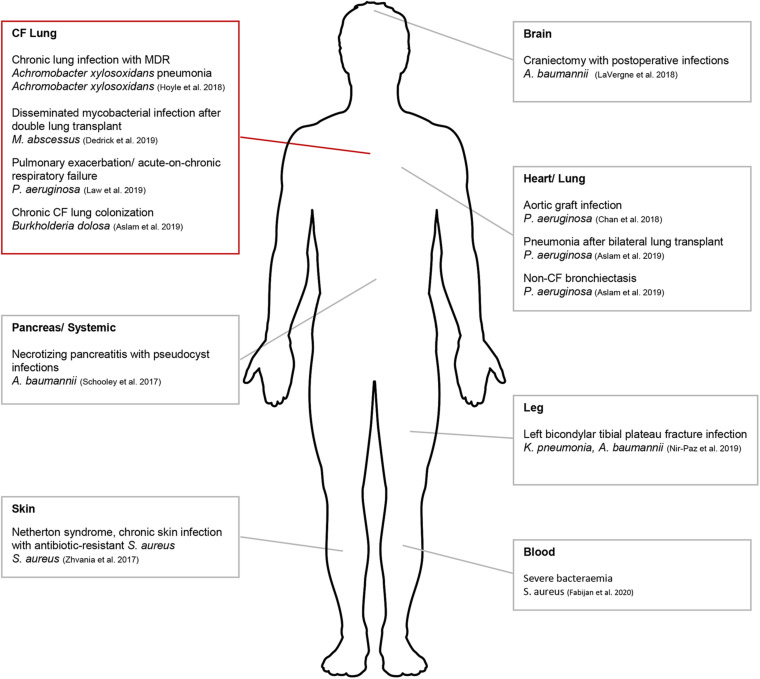
Schematic diagram indicating areas where phage therapy had been applied clinically for compassionate use. Compassionate use in CF denoted by red box. This figure was created using Servier Medical Art templates, which are licensed under a Creative Commons Attribution 3.0 Unported License; https://smart.servier.com.

In the West, hesitancy in translating to a human treatment pipeline has been primarily due to the lack of published scientific reports and rigorous safety data. However, human phage therapy has gained significant traction in the last 5 years and has been tested clinically (Figure 1). One study used phage therapy to treat a patient who had been infected with MDR *Acinetobacter baumanii* (*A. baumanii*) (Schooley et al., 2017) (Figure 1). Multiple rounds of antibiotic treatments had initially failed to control the infection which had spread beyond the abdominal cavity. *In vitro* experiments were initially conducted to determine phage specificity and efficacy against the infective strain and approval for clinical use was then sought from the Food and Drug Administration (FDA). This was granted on compassionate grounds and treatment resulted in the patient’s full recovery (Schooley et al., 2017). Since this pioneering study, compassionate ground use of phage therapy has been successfully used to treat patients with distinct MDR infections (Chan et al., 2018; Dedrick et al., 2019). A recent Australian study has highlighted the translatable potential of phage therapy into clinical applications to treat multiple patients suffering from severe *Staphylococcus aureus* (*S. aureus*) bacteremia (Petrovic Fabijan et al., 2020b). While this specific study supported phage therapy for disseminated bacterial infection, what is less clear is whether it is applicable for patients suffering from chronic soft tissue infection caused by biofilm-producing bacteria.

## The Potential of Phage Therapy in CF

In order to more accurately assess how phage therapy is currently advancing, we conducted an initial review of articles in NCBI using the article title search term “Bacteriophage.” We selectively excluded all published abstracts and still identified >12,000 published articles in this area. When “Therapy” was included in the article title search term, 141 published articles were found, including 46 review papers. The remaining 95 original research papers were then screened and found to report on research performed in the medical (83) or veterinary sphere (12). Collectively, these findings imply that despite an enormous amount of research being carried out in the discovery and characterization of phages, the field has struggled to translate results into standard clinical care. Although successful use of phage therapy in CF has been reported, this has been approved on compassionate grounds and its translation into standard clinical care still requires additional rigorous, systematic and detailed exploration (Supplementary Table 1).

While *P. aeruginosa*, *S. aureus*, and *Burkholderia cepacia* complex (*B. cepacia* complex) are commonly isolated pathogens from individuals with CF, *Mycobacterium* and other infections are emerging. One ideal therapeutic target is *Mycobacterium abscessus* (*Mab*), an emerging pathogen isolated from the lungs of individuals with CF (Bernut et al., 2019). *Mab* is able to form biofilms (Qvist et al., 2015; Fennelly et al., 2016) and is intrinsically resistant to many antitubercular drugs, requiring prolong usage of at least three antimicrobial drugs for up to 2 years (Floto et al., 2016). While there has been clinical evidence correlating *Mab* infections with declining lung function, whether the bacterium is the causative agent is unknown (Sanguinetti et al., 2001; Esther et al., 2010; Qvist et al., 2016). The first reported compassionate use of phage therapy in CF was for a 15-year old patient with disseminated *Mab* infection at sites other than the lung following bilateral lung transplant (Dedrick et al., 2019; Figure 1). Despite additional antimicrobials used, *Mab* had infected other areas of the body apart from the surgical site. A cocktail of three phages was used and were bioengineered from mutant derivatives that displayed optimum lysis of *Mab* isolated directly from the patient. A single topical application was followed by intravenous therapy for ∼32 weeks. Upon completion, lung function had improved from 50% FEV_1_, immediately following transplant up to 80–90% FEV_1_. The significance of this study lies in the outlined pipeline needed to ensure phage therapy translation including appropriate *in vitro* safety and efficacy studies.

The second reported compassionate use of phage therapy in CF was for a 26-year old patient who presented with severe acute-on-chronic respiratory failure resulting in mechanical ventilation (Law et al., 2019). The patient was infected with two strains of MDR *P. aeruginosa* and received multiple courses of high dose antibiotics which then triggered kidney failure. Approval for AP-PA01, a cocktail of four bacteriophages produced by AmpliPhi Biosciences Corporation (now Armata Pharmaceuticals) was granted and phages were administered intravenously. After 8-weeks of treatment, the patient was successfully cleared of *P. aeruginosa* colonization without side effect, was ambulatory and stable enough to be once again placed on a lung transplantation waitlist. Other pathogens targeted by phage therapy in CF have included *Achromobacter xylosoxidans* and *Burkholderia dolosa* (Figure 1), due to increasing incidences of multidrug resistance (Kalish et al., 2006; Jeukens et al., 2017). Phage therapy has also been applied for compassionate use at various sites other than the lung (non-CF associated) suggesting that a number of other CF-associated pathogens including *S. aureus*, *A. baumanii*, and *Klebsiella pneumoniae* may also be clinically targeted using phage therapy (Figure 1). Emerging CF pathogens such as *Stenotrophomonas maltophilia*, are also of concern due to their increasing incidence of isolation (Millar et al., 2009; Razvi et al., 2009; Emerson et al., 2010) and multidrug resistance (Gajdács and Urbán, 2019; Gröschel et al., 2020). Since their infectivity and transmission mechanisms are still to be elucidated (Stanojevic et al., 2013; Gröschel et al., 2020) which would typically direct targeted treatment regimens, phage therapy has been postulated as a potential therapeutic option currently (Chang et al., 2005; Peters et al., 2015, 2017).

There have also been attempts to assess phage therapy as part of potential standard clinical care, however, rarely have results of performed clinical trials been published. It is critical to report this information in order to inform the trial process and guide future study designs. An example identified through a screen of registered trials on ClinicalTrials.gov is “MUCOPHAGES” (NCT01818206). Although identified as completed, there is no available information on formulation used, length of phage exposure, and importantly what bacteria were being targeted. Closed findings only act to hinder progress and there needs to be greater transparency if we are to pipeline this therapy into standard clinical care.

Another challenge is that most published studies on the efficacy of phage therapy on CF-derived pathogens have been performed using pathogens in planktonic state. However, bacterial pathogens often exist in biofilm state within the CF airways. Such significant differences between the *in vitro* experiment model and *in vivo* CF airway conditions render the translation of benchside result to bedside questionable. Biofilms are made up by a pure population or a consortium of microorganisms, creating a unique bacterial lifestyle and niche habitat, enhancing protection against antimicrobials, and able to establish in conditions with a flow of liquid and withstand shear force, forming classic tower-like structures (Flemming and Wingender, 2010; Flemming et al., 2016). Thus, phages with effective biofilm dispersal capabilities are highly desirable. Current evaluation of phage activity on biofilm is usually performed on abiotic surfaces which typically do not account for biological flow strength, nor the properties of the tissues where typically biofilms reside, namely (in the case of CF) the airway epithelium. Flow-cell systems and fluorescence microscopy are considered the “gold standard” to observe spatiotemporal changes of biofilm heterogeneity in real-time (Crusz et al., 2012; Tolker-Nielsen and Sternberg, 2014; Haagensen et al., 2015). The use of flow-cell also maintains biofilm’s viability while the hydrodyanamic movement removes planktonic bacteria and eliminates a confounding factor in the measurement of phage therapy efficacy on the biofilms. Thus, having a biological relevant model of CF would assist in our understanding not only of biofilm formation but also of bacterial evasion of the host innate and adaptive immune responses.

### Phage Therapy: CF Experimental Models and What Is Missing

While lung disease is the major cause of morbidity and mortality in CF, there are currently no effective models that mimic the CF lung infection pathology (Table 1). Despite different animal models developed to study CF, the data generated often are still limited by the models. The CF porcine model successfully mimics several CF manifestations including meconium ileus (MI), pancreatic deficiency and subsequent gastrointestinal tract obstruction with similar airway epithelia and submucosal gland (SMG) activities at birth to humans (Rogers et al., 2008). However, the model is not commonly used due to severe disease pathology and difficulties in maintaining longevity of the pigs due to MI. High animal husbandry costs also make the model unfeasible for most laboratories to utilize. CF murine and rodent models have also been explored to mimic the pathophysiological states of CF *in vivo*. The CF mouse model was developed shortly after the discovery of the CFTR gene (Snouwaert et al., 1992) where it successfully mimicked chronic lung infection with mucoid strains of *P. aeruginosa* (Coleman et al., 2003). However, differences between the abundance and distribution of airway epithelial cell (AEC) types make the model less translatable to the human CF airway (Plopper et al., 1983; Grubb and Boucher, 1999). In comparison, rats have more developed SMGs and their implication in CF lung pathology is well studied and linked with disease progression in humans (Smolich et al., 1978; Jayaraman et al., 2001; Wine, 2004; Tuggle et al., 2014). Mucus plugging, one of the characteristics of CF lung disease has been observed in the lungs of a *Cftr^–/–^* rat model developed by Birket et al. (2018). This is an essential requirement when studying mucociliary transport and bacterial colonization (Birket et al., 2018). Chronic infections are also difficult to establish in these models and typically use bacterial cells embedded into agar beads before lung installation (Bragonzi et al., 2009; Facchini et al., 2014; Kukavica-Ibrulj et al., 2014; Kukavica-Ibrulj and Levesque, 2015; Bayes et al., 2016; Cigana et al., 2016, 2020). Results are also hard to interpret since it is difficult to distinguish induced immune response initiated by bacteria such as *P. aeruginosa* and the presence of a foreign body (agar beads). Finally, the *Cftr*^–/^*^–^* ferret model resulted due to the high similarity in the anatomy and biology of its lungs with those of humans (Sun et al., 2008). The CF ferret model mimics the human condition in its susceptibility to lung infection, and lung function decline as the main cause of mortality (Sun et al., 2010, 2014). Despite this model being the best to test therapeutics against lung infection and inflammation, progress in this field remains slow as inconsistencies in severity of lung disease confound the ability to understand the impact of disease progression and efficacy of therapeutics.

**TABLE 1:** Comparison of available CF animal models.

Animal	Advantages	Disadvantages
Pig	• High genetic and anatomic similarity of organs (Cooper et al., 2008; Längin et al., 2018; Sykes and Sachs, 2019).	• Expensive husbandry (Ostedgaard et al., 2011).
	• Highly similar disease pathology with humans (Meyerholz et al., 2010).	• Requires large animal facility.
	• Comparable immunological response to infections.	• Deaths soon after birth due to meconium ileum (MI) (Rogers et al., 2008).
Mouse	• Chronic lung infection with mucoid strains of *P. aeruginosa* (Coleman et al., 2003).	• Submucosal gland (SMG) present only in proximal trachea (Colledge et al., 1995; Hikke Van Doorninck et al., 1995; Zeiher et al., 1995).
	• Similar lung physiological structure after infection.	• Do not display early CF lung phenotype
		• Less severe disease pathology in the lungs
		• Inconsistencies in animal husbandry, strains of *P. aeruginosa* used (Morissette et al., 1995; Coleman et al., 2003; Bragonzi et al., 2005; Hoffmann et al., 2005).
		• Host response mechanisms different from human CF airway epithelia.
Rat	• Extensive SMGs in airways (Smolich et al., 1978; Jayaraman et al., 2001; Wine, 2004; Tuggle et al., 2014).	• Longitudinal study of KO rats required.
	• Cost-efficient husbandry.	• Stability of *Cftr* gene deletion in rats not reported.
	• Shorter gestation period.	• Fairly new model which requires more investigation toward lung infection with bacteria.
	• Mucus plugging observed, impaired mucociliary transport (Birket et al., 2018).	• Airway disease phenotype yet to be elucidated.
Ferret	• High similarity to human lungs both anatomically and biologically (Darnell et al., 2007).	• Varying degrees of spontaneous lung disease.
	• Extensive SMGs (Smolich et al., 1978; Engelhardt et al., 1992; Sehgal et al., 1996; Wine, 2004).	• Difficult to track lung disease progression (Sun et al., 2010, 2014).
	• Exhibits characteristic responses of human CF lungs to bacterial infections.	• No consistent predominant bacteria isolated from lung microbiome (influenced by the gut microbiome).
	• Shorter gestation time (4–6 months) in comparison to the porcine model.	• Lack of sodium epithelial channel (ENaC) dysregulation.
	• Less costly animal husbandry and smaller animal facility required.	• More expensive animal husbandry than rodent model, limiting wide availability.
Zebrafish	• Low expense maintenance.	• Lacks mammalian organs.
	• Fluorescence tracking *in vivo* (Cafora et al., 2019).	
	• High reproduction rate.	

### Cell Cultures and the Role They Play in Phage Therapy Research

An alternative to the use of animal models is the use of AEC cultures. Universal cell lines such as H441 (lung epithelial cells) (Hermanns et al., 2004) and HeLa (cervical carcinoma cells) (Scherer et al., 1953; Lucey et al., 2009) have been utilized in a wide variety of research ranging from cancer to infection models. Immortalized CF cell lines such as CFBE45o – (CF bronchial epithelial cells) (Gruenert et al., 2004) and CuFi (Zabner et al., 2003) have also been instrumental in different studies including the development and efficacy testing of therapies, both genetic and pharmacological. AEC lines have also been valuable in high-throughput screening of potential drugs. However, submerged monolayer cultures of CF AEC lines are unable to mimic the microenvironment of a CF lung, which is characterized by mucus production and persistent inflammation status. Another limitation of CF AEC lines is the inability to test for interactions and efficacy of phage therapy when used in conjunction with CFTR modulators that target specific mutations in the CFTR gene. However, the use of primary AECs obtained from patients would be able to overcome this limitation. Although CF AEC lines can be grown at air-liquid interface (ALI), there have been inconsistencies in barrier integrity, as well as their capacity to form the multiple cell layers that comprise the airway lining architecture. Furthermore, it is unknown whether they also produce multiple cell types typically including goblet cells (Hermanns et al., 2004; Ren et al., 2016).

Fully differentiated primary AECs have been described extensively in the literature and serve as the most representative *in vitro* model of the airway (Randell et al., 2011; Martinovich et al., 2017). Although AEC ALI cultures may not mimic the biological environment perfectly, they demonstrate features of the airway, with polarization of progenitor cells into ciliated, basal, undifferentiated columnar and secretory cells. Tight junctions are also well developed in 3D ALI cultures (Crystal et al., 2008; Choi et al., 2016; Looi et al., 2016, 2018). A concerted effort by the Australian Respiratory Early Surveillance Team for Cystic Fibrosis (AREST CF) to biobank AECs collected from children with CF longitudinally has created a valuable repository that is readily accessible (Sutanto et al., 2011; Garratt et al., 2017). With prospective sampling, an *in vitro* AEC ALI model could be built in the laboratory to screen for and test efficacies of phages against clinical isolates from CF patients, accounting for immunological responses arising from the application of phages. Currently, there are no appropriate biofilm models grown on AECs described in the literature at the time of writing this review (2020). While most research on medical biofilms has been carried out on abiotic surfaces (Alemayehu et al., 2012; Tinoco et al., 2016) or submerged monolayers of AEC (Trend et al., 2018), these are not reflective biological representations of the complex airway epithelium where biofilms establish. Despite advances in cell culture methodologies, recapitulating the entire human lungs remains a challenge. The human lung is a complex organ with more than 59 cell types located in different anatomical locations (Travaglini et al., 2019) and compounding this further, is the complex genotypic and molecular mechanisms within and between these cells. However, a laboratory model is still essential for airway phage therapy application and AEC ALI cultures still remain the most relevant model that mimics key features of the airway epithelium. Recent developments in 3D printing of organs is rapidly evolving and may be able to add further complexity whilst remaining accurate in its recapitulation of the human lung (Grigoryan et al., 2019; Shrestha et al., 2019).

Delivery of prepared phages could also be tested on AEC ALI cultures to capture a more comprehensive pre and post application reaction of the host mammalian cells (Buckley et al., 2018). While topical and/or intravenous applications of delivery have been effective, they are not ideal for pulmonary infections, particularly in pediatrics. Compliance toward the therapy is an important factor that would ensure optimum efficiency of released phages in the lungs against the pathogens. Therefore, inhalation of phages would be the most suitable for pulmonary infections (Sahota et al., 2015; Malik et al., 2017; Wallin et al., 2019). Currently, studies measuring the stability and efficacy of both liquid (Carrigy et al., 2017) and dry powder formulations (Chang et al., 2017, 2020) of nebulized phages have been conducted and show potential in ensuring the dispersion into lower airway of the lungs. Further work is needed to fully elucidate the best formulation and delivery methodologies before phage therapy can be implemented as part of standard clinical care.

## Phages and Antimicrobials: Safety and the Partnership Potential

Safety and side effects of phage therapy remain the greatest concerns in the translation to clinical care. To date, there have been no known adverse effects or mild effects that failed to resolve by the end of the treatment reported from the application of phages (Vandenheuvel et al., 2015; Supplementary Table 1). Although there have been clinical trials that had been terminated, this was due to a lack of significant improvement (Sarker et al., 2016) or insufficient efficacy (Jault et al., 2019). While animal models do not fully mimic the human’s immunological response, Trend et al. (2018) have demonstrated that the application of *P. aeruginosa*-specific phages on primary AECs did not elicit an immunological response (Trend et al., 2018). Furthermore, although Żaczek and colleagues reported that anti-staphylococcal phages applied orally or locally did induce a humoral response in some patients, there was no increase in inflammatory markers or reduction in effectiveness of phages (Żaczek et al., 2016). Safety and tolerability have also been demonstrated by two recent studies using phage therapy to treat *S. aureus* chronic rhinosinusitis (Ooi et al., 2019) and severe sepsis (Petrovic Fabijan et al., 2020b), respectively. In cases of phage therapy directed against *P. aeruginosa* respiratory infections, no adverse phage therapy-related effects have yet been identified (Aslam et al., 2019; Law et al., 2019). Nevertheless, interpretation of potential safety and side effects of phage therapy has been limited by the lack of published results.

Currently, approval for phage therapy in CF has been granted on compassionate grounds. However, translation of phage therapy to standard clinical care would most likely target a larger population of patients infected with antibiotic susceptible pathogens. Those on prophylaxis and prolonged treatment regimes would benefit the most since *P. aeruginosa* isolated from early CF lungs of children have been found to be more similar to environmental strains and susceptible to antimicrobial treatments (Burns et al., 2001; Jelsbak et al., 2007; Marvig et al., 2015). A combination of phage therapy may act synergistically with antimicrobials to potentiate the reduction or delay of MDR infection occurrence (Knezevic and Aleksic Sabo, 2019; Petrovic Fabijan et al., 2020a). Phage therapy in combination with suboptimal concentrations of antibiotics has shown potential in eradicating infection more efficiently, lowering the risk of adverse effects from long-term usage of antibiotics (Kirby, 2012; Knezevic et al., 2013; Kamal and Dennis, 2015). A study by Chan et al. (2016) demonstrated that the application of φOMKO1 was able to restore antibiotic sensitivity in *P. aeruginosa*, which reinforces the potential of phages against drug-resistance (Chan et al., 2016). A further benefit of joint use of antibiotics and phage therapy may be a reduction in the development of bacterial resistance to phages, as even when phage mixtures (cocktails) are employed (Wright et al., 2019), gain of phage resistance can occur throughout the length of therapy (Schooley et al., 2017). Further examples of this approach have recently been reviewed by Tagliaferri and colleagues (Tagliaferri et al., 2019). The potential of phage therapy when used in combination with antimicrobials is yet to be fully exploited and future detailed exploration in this area is warranted.

## Phage Therapy: Future Prospects

The future of phage therapy is not necessarily to replace current therapies, rather there is potential for clinical applications to supplement and provide an alternative treatment for infections. With a predicted shift into personalized phage therapy in the immediate future, research in this area is likely to grow at an exponential rate (Pirnay, 2020). However, the full potential of phage therapy can only be achieved when there is transparency and a willingness to share knowledge as well as resources. Ideally, phage libraries should be freely available through a network of collaboration, and information on preparation and delivery methods for phages meant for clinical usage should be well documented. Phage formulation and delivery are also critical considerations in order to direct activity to targeted areas and maximize efficacy. In fact, use of phage therapy already appears to be coordinated in various countries according to national regulations, and by major public health institutes such as Therapeutic Goods Administration (TGA) (Australia) (Donovan, 2017; Lin et al., 2019), Food and Drug Authority (FDA) (United States of America) (Jarow et al., 2017; Puthumana et al., 2018) and the European Medicines Agency (EMA) (Europe) (Balasubramanian et al., 2016; Debarbieux et al., 2016). Importantly, a universal code of ethics should be established and regulatory bodies reach a consensus on the exchange of information, usage of phages as treatment and reporting of treatment outcomes (Furfaro et al., 2018; Borysowski et al., 2019). Due to the critical nature of the rise of MDR, increasing the urgency for phage therapy to be implemented as standard care, alternative therapies to be translated into clinical applications need to be expedited. A concerted effort with both local and global partners could see phage therapy being translated into standard care in the next 5 years.

## Author Contributions

RN and AK conceived the review topic and focus. RN conducted literature review and drafted the manuscript. AK, AT, BC, and SS contributed to the structure and content, provided critical review and approved the final version to be published. All authors contributed to the article and approved the submitted version.

## Conflict of Interest

The authors declare that the research was conducted in the absence of any commercial or financial relationships that could be construed as a potential conflict of interest.
