# Supplementary material for: Overcoming Challenges to Make Bacteriophage Therapy Standard Clinical Treatment Practice for Cystic Fibrosis
Source: Front Microbiol. 2021 Jan 11;11:593988. doi: 10.3389/fmicb.2020.593988 (PMC7829477; doi:10.3389/fmicb.2020.593988)
Supplement: Supplementary file 1 [file Table_1.docx]

Supplementary Material

Supplementary Table 1. Phage therapy studies related to CF and/or compassionate use in the past 15 years

| Phages used | Bacterial species | Clinical presentations/ complications | Singular/ cocktail preparation | Clinical outcome(s) | Reference |
| --- | --- | --- | --- | --- | --- |
| - Biophage-PA (BC-BP-01, BC-BP-02, BC-BP-03, BC-BP-04, BC-BP-05, BC-BP-06) | *Pseudomonas aeruginosa*^#^ | Chronic otitis with antibiotic-resistant *P. aeruginosa* | Cocktail | 3 out of 12 patients had <10% *P. aeruginosa* load as compared to day 1 | (Wright et al. 2009) |
| - BFC-1 (14/1, PNM, ISP) | *Staphylococcus aureus*  *Pseudomonas aeruginosa*^#^ | Infected burn wound | Cocktail | Bacterial load remained unchanged after application of BFC-1 or standard treatment, no adverse results observed from eight patients | (Merabishvili et al. 2009; Rose et al. 2014) |
| - WPP-201 | *Pseudomonas aeruginosa*  *Staphylococcus aureus*  *Escherichia coli*^#^ | Chronic venous leg ulcer | Cocktail | No significant improved healing rate nor frequency | (Rhoads et al. 2009) |
| - PAK-P1 | *Pseudomonas aeruginosa*** | Acute lung infection in mouse model | Singular | 100% survival with phage-to-bacteria ratio of 10:1 and 1:1. 80% survival with phage-to-bacteria ratio of 1:1 in second independent experiment | (Debarbieux et al. 2010) |
| - PAK_P3 - Pc_CHA | *Pseudomonas aeruginosa*** | Acute lung infection in mouse model | Singular | Improved survival and reduced bacterial counts over two magnitude | (Morello et al. 2011) |
| - Pyobacteriophage - *Staphylococcus* bacteriophage - Fersis | *Staphylococcus aureus*^+^ | Netherton syndrome, chronic skin infection with antibiotic-resistant *S. aureus* | Cocktail | Skin regeneration and no hospitalization required for general infection 6 months after starting on phage therapy | (Zhvania et al. 2017) |
| - PAK_P1 - PAK_P2 - PAK_P3 - PAK_P4 - PAK_P5 - P3_CHA - CHA_P1 - PhiKZ - LUZ19 - LBL3 | *Pseudomonas aeruginosa*** | CF clinical *P. aeruginosa* suspended in 58 sputum samples collected from 58 CF patients | Cocktail | 35.1% coverage of cocktail against all *P. aeruginosa* isolated from 58 CF patients | (Saussereau et al. 2014) |
| - T4-like (AB2, 4, 6, 11, 46, 50, 55; JS34, 37, 98, D1.4) | *Escherichia coli*^#^ | *E. coli* diarrhea | Cocktail | Clinical trial was terminated as there was no significant improvement between treatment with T4-like, Microgen ColiProteus phage cocktail and placebo | (Sarker et al. 2016) |
| - PELP20 | *Pseudomonas aeruginosa* (LESB65 and LESB65 host-adapted derivative strain NP22_2)** | Chronic lung infection in mouse model | Singular | 3-log reduction in artificial sputum medium biofilm model and complete clearance of *P. aeruginosa* from mouse lungs | (Waters et al. 2017) |
| - **ϕPC** (AC4, C1P12, C2P21, C2P24) - **ϕIV** (AB-Navy1, AB-Navy4, AB-Navy71, AB-Navy97) - **ϕIVB** (AB-Navy71, AbTP3ϕ1) | *Acinetobacter baumannii*^+^ | Necrotizing pancreatic with pseudocyst infections with MDR *A. baumannii* | Cocktail | Full recovery | (Schooley et al. 2017) |
| - PYO2 - DEV - E215 - E217 - PAK_P1 - PAK_P4 | *Pseudomonas aeruginosa*** (CF-derived *P. aeruginosa* used to isolate novel phages) | Acute respiratory infection in mice and bacteraemia in wax moth (*Galleria mellonella*) | Cocktail | Reduction of bacterial burden in lungs and 100% survival rate of infected mice, increased survival rate post-infection | (Forti et al. 2018) |
| - Unpublished | *Acinetobacter baumannii*^+^ | Craniectomy with postoperative infections with MDR *A. baumannii* | Unpublished | Patient expired | (LaVergne et al. 2018) |
| - OMKO1 | *Pseudomonas aeruginosa*^+^ | Aortic graft infection with MDR *P. aeruginosa* | Singular | No further evidence of bacterial infection | (Chan et al. 2018) |
| - *Achromobacter* phages | *Achromobacter xylosoxidans** | Chronic lung infection with MDR *A. xylosoxidans* pneumonia | Cocktail | Improved lung function (FEV_1_), reduction of cough and resolution of dyspnea | (Hoyle et al. 2018) |
| - Muddy - BPs33ΔHTH-HRM10 - ZoeJΔ45 | *Mycobacterium abscessus** | Disseminated mycobacterial infection after double lung transplant in CF patient | Cocktail | Full recovery | (Dedrick et al. 2019) |
| - AB-PA01 | *Pseudomonas aeruginosa** | Pulmonary exacerbation that further deteriorated to acute-on-chronic respiratory failure | Cocktail | Full recovery | (Law et al. 2019) |
| - AB-PA01 - AB-PA01 m1 - Navy cocktail 1 - Navy cocktail 2 | *Pseudomonas aeruginosa^+^* | Post bilateral lung transplant complication, multiple episodes of pneumonia | Cocktail (in combination with antibiotics) | No adverse effect recorded and patient discharged from hospital | (Aslam et al. 2019) |
| - AB-PA01 | *Pseudomonas aeruginosa*^+^ | Non-CF bronchiectasis, recurrent MDR *P. aeruginosa* infections post- lung transplant | Cocktail (in combination with antibiotics) | No adverse effect recorded and patient discharged with colistin for suppressive therapy |  |
| - BdPF16phi4281 | *Burkholderia dolosa** | *B. dolosa* colonization post-bilateral lung transplant, recurrent *B. dolosa* pneumonia | Singular (in combination with antibiotics) | No adverse effect recorded from phage therapy, patient expired due to clinical deterioration) |  |
| - φAbKT21phi3 - φKpKT21phi1 | *Klebsiella pneumonia, Acinetobacter baumannii*^+^ | Left bicondylar tibial plateau fracture infected with MDR *K. pneumonia* and XDR *A. baumannii* | Cocktail (in combination with antibiotics) | Full recovery | (Nir-Paz et al. 2019) |
| - PP1131 - (PhagoBurn) | *Pseudomonas aeruginosa*^#^ | Burn wound infections | Cocktail | Trial stopped due to insufficient efficacy, PP1131 decreased bacterial burden in wounds slower than standard of care | (Jault et al. 2019) |
| - Unknown | *Klebsiella pneumonia^#^* | Chronic relapsing urinary tract infection after renal transplant | Not reported (bacteriophage suspension obtained from Eliava Institute) | Urethritis symptoms resolved (treated in combination with meropenem) | (Kuipers et al. 2019) |
| - AB-SA01 | *Staphylococcus aureus*^+^ | Severe bacteraemia (predicted average six-month mortality rate at 44%) | Cocktail | Seven out of 13 patients survived past 90 days | (Petrovic Fabijan et al. 2020) |
| - AB-SA01 | *Staphylococcus aureus* | Chronic rhinosinusitis | Cocktail | 2 out of 9 recruited patients had eradication of infection | (Ooi et al. 2019) |
| - EcoActive   (NCT03808103) | *Escherichia coli*^#^ | Adherent Invasive *Escherichia coli* (AIEC) in Crohn’s Disease | Cocktail | Currently recruiting | <https://clinicaltrials.gov/ct2/show/NCT03808103> |
| - NCT04287478 | *Klebsiella pneumonia,*  *Escherichia coli*^#^ | Urinary tract infections | Cocktail | Not yet recruiting | <https://clinicaltrials.gov/ct2/show/NCT04287478?term=phage+therapy&draw=2&rank=2> |

(* - Compassionate use on CF patient, ** - Report using CF-derived bacterial samples, # - clinical trial, + - Compassionate use)
